# Aids to Nursing

**Published:** 1888-02-18

**Authors:** M. M Basil

**Affiliations:** Author of "The Commoner Accidents to Life and Limb"


### Aids to Nursing.

By M. M. Basil, M.A., M.B., C.M., Author of "The Commoner Accidents to Life and Limb."

(*Continued from p. 332.*)

We must consider the warming of the sick-room in immediate relationship with its ventilation, as in this climate it is necessary to secure them in combinafion with each other. The main cause here of defective ventilation is the difficulty of maintaining the proper degree of warmth, with due renewal of air, consistently with rigid economy. As a general rule the sick-room should have a tolerably uniform temperature, determined from time to time by a thermometer, placed, not in proximity to the fire, nor exposed to its direct rays, nor hanging from the gas-burner, but situated somewhere about the middle of the room. Dr. Sankey has shown that to obtain the exact temperature of a room the bulb of the thermometer must be guarded against direct radiation, even from a distant source of heat. The temperature should generally be kept at 60 deg. to 62 deg. F.; in cases of children with measles, at 65 deg. F.; and when nursing babies and very old patients, at 70 deg. or even higher. It should not be allowed to sink lower than 55 deg. F. The teaching on this last point, however, in most books, especially text-books on nursing, is open to grave doubts. "There are many diseases," says Dr. Parkes, "greatly benefited by a low temperature, especially all those with preternatural heat. It applies, almost without exception (scarlet fever?), to the febrile cases in the acute stage, that it is desirable to have the temperature of the air as low as 50 deg., or even 45 deg. or 40 deg. Cold air moving over the body is a cooling agent of great power, second only, if second, to cold affusion; nor is there danger of bad results if the movement is not too great. The Austrian experiments on tent hospitals show conclusively that even considerable cold is well borne. Even in the acute lung affections this is the case. Pneumonia cases do best in cold wards, provided there is no great current of air over them. Many cases of phthisis bear cool air, and even transitions of temperature, well, provided there be no great movement of air. On the other hand, it would appear that chronic heart diseases and lung congestion, emphysema of the lungs, and diseases of the same class require a warm air, and perhaps a moist one. With respect to the inflammatory affections of the throat, larynx, and trachea, no decided evidence exists; but the spasmodic affections of both larynx and bronchial tubes seem benefited by warmth."

The dread of chilling by too free a ventilation patients who are securely tucked in under blankets. is usually groundless, still one cannot insist too strongly upon the necessity of regularly watching the thermometer in the room. In no case of serious disease should you omit to observe the temperature of the room and of your patient between midnight and daylight. The small hours of the morning, the "Hours of the Fates" of the Ancients, are the most critical ones to the sick, and often the flickering light is extinguished by a sudden lowering of atmospheric temperature. "Sank early on the morning of —— day" would be the concluding sentence in the life history of 70 per cent. of men, women, and children. In nearly all diseases the patient's temperature attains its highest point before midnight, and after that period there is often a sudden fall. So constant, indeed, is this fall of temperature in disease as well as in health, that the reverse condition, viz., a rise of temperature after midnight, is generally of ill omen in the sick-room. It is, therefore, of extreme importance that by attention to the thermometer, by feeling the patient's extremities, by observing the pinched or blue condition of the face or ears, by noticing increased slowness of speech, the nurse should be forewarned of serious danger and at once forearmed against it. An extra coverlet, a little warm food, a hot bottle applied to the feet, a kettle simmering on the hob, will often ward off a fatal issue, and not only delay death but save a good life. The dread of the "east wind" often means the absence of a good nurse or the presence of one with a headpiece of little more value than the trimming of the cap covering it. A similar state of semi-collapse may occur not only through the night but at any hour in the day, by a sudden fall of temperature after prolonged fever, and you must be on your guard to foresee danger and ward it off.

Most patients who are perfectly safe from chills while in bed run great dangers in this respect during convalescence. You should be especially on your guard after scarlet fever, measles, whooping cough, and parturition; in cases of burns and scalds, in small-pox, in all cases of severe diarrhœa, even when caused by a smart purgative, or prolonged discharges of any form, and in all cases where your patient is very anxious or nervous, for example, before and after operations. Then, again, you must especially guard against cold feet in all cases of pelvic disease in the female, and a similar precaution is necessary in the male with bladder or urethral disease.

In bathing, and in dressing and undressing all convalescents, you must carefully guard against draughts. The change of linen must be properly and uniformly warmed before being put on. No portion of it should be made so hot as to cause a shock to a sensitive patient.

All patients who are paralysed, insensible, drunk, or narcotised, very stupid or very old, must be watched as to their temperature, as they may be seriously chilled without taking any steps to avoid danger, or they may be roasted by sitting too close to the fire for a prolonged period. Many cases are now recorded in which rapidly destructive disease of the knee joints has followed prolonged exposure before a large fire.

As a rule, you must cover your patient's body and limbs suitably to the season, and insist upon the head being uncovered. That the head should not be kept under blankets is the general rule. In certain cases, however, you must make temporary exceptions. If the construction of the room will not allow you to secure ventilation and warmth combined, you must from time to time cover up your patient, admit fresh air into the room and uncover the head after the temperature of the room has nearly reached its normal point. This precaution is especially necessary in certain diseases; such are all cases of severe sore throats, including croup, mumps and diphtheria: scarlet fever and measles, bronchitis and pneumonia, and, lastly, in all fevers, especially typhoid fever, with throat complications.

Hot bottles are a subsidiary means of securing warmth, and may be considered here. The best of the kind are stone ones. Indiarubber has an objectionable smell when hot, and gets readily out of order. Tin foot-warmers require great care as to their temperature. In an emergency any well-corked bottle can be made to answer the purpose. You must be sure that your patient, especially if paralysed, runs no risk of being burnt. The bottles used should be covered, thoroughly corked so that no leaking will be possible under any provocation by kicking and tumbling about, and should be changed every eight hours, or sooner if they are felt to be cold. If applied to stumps after operations, they must be removed as soon as the patient has rallied from the shock, otherwise they tend to increase the chances of bleeding. Similarly, it must be remembered that in case of pelvic bleeding their use to the feet will tend to increase the bleeding.

In general, you must depend upon an open fireplace as your main agent in warming the sick-room. It is also an efficient means of ventilation, and should be secured in every case of serious disease. Certain elementary principles regarding it must, however, be steadily kept in mind. It should not be made into a fierce furnace to roast your patient at for a couple of hours, and then allowed to die out. Its size must be regulated in accordance with the weather, the hour of the day, and the degree of heat required in the room. Many a patient has verily to go through a fiery trial about three to nine p.m., when probably he is burning with a high febrile temperature, and as if to make up for this hardship, he has to shiver for an equal number of hours early in the morning. This process of hardening a patient by plunging him several times from a torrid into a frigid zone may have its use in the way of sifting out what amount of Christian patience there is in him, but it certainly is not conducive to health.

In wards where children, epileptic patients, or very aged people are nursed, the fireplace should be guarded.

Lastly, use as little gas as you can in the sick-room. Miss Nightingale states that one gas-jet consumes as much air as nine men do. This is very much to be doubted; at any rate, in point of unhealthiness, to have three gas-jets in a room does not mean having twenty-seven men living in it. The products of combustion in the two cases are not the same, though for the sake of convenience the amount of carbonic acid set free is taken as the measure of impurity in each case. Still, however, the general fact that the combustion of gas in a room is a great cause of unhealthiness holds good, and the less of it we have the better for all parties. Burning several jets of gas to warm a sick-room is not the work of any sensible human being. It is a ruinously costly means of securing warmth, ruinous to the pocket and health in the long and in the short run, and in every conceivable sort of run. It is, however, by no means quite a thing of the past, as one has repeatedly known of grave old doctors, who seemed almost to believe that wisdom would die with them, directing several jets of gas to be burnt for many hours to heat a room. You should not be responsible for such an action, except when obeying orders whose wisdom is beyond questioning.

*(To be continued.)*

